# Molecular Genetic Analysis of Stomach Contents Reveals Wild Atlantic Cod Feeding on Piscine Reovirus (PRV) Infected Atlantic Salmon Originating from a Commercial Fish Farm

**DOI:** 10.1371/journal.pone.0060924

**Published:** 2013-04-19

**Authors:** Kevin Alan Glover, Anne Grete Eide Sørvik, Egil Karlsbakk, Zhiwei Zhang, Øystein Skaala

**Affiliations:** 1 Institute of Marine Research, Bergen, Norway; 2 Jiangsu Institute of Marine Fisheries, NanTong City, P. R. China; University of Toronto, Canada

## Abstract

In March 2012, fishermen operating in a fjord in Northern Norway reported catching Atlantic cod, a native fish forming an economically important marine fishery in this region, with unusual prey in their stomachs. It was speculated that these could be Atlantic salmon, which is not typical prey for cod at this time of the year in the coastal zone. These observations were therefore reported to the Norwegian Directorate of Fisheries as a suspected interaction between a local fish farm and this commercial fishery. Statistical analyses of genetic data from 17 microsatellite markers genotyped on 36 partially-degraded prey, samples of salmon from a local fish farm, and samples from the nearest wild population permitted the following conclusions: 1. The prey were Atlantic salmon, 2. These salmon did not originate from the local wild population, and 3. The local farm was the most probable source of these prey. Additional tests demonstrated that 21 of the 36 prey were infected with piscine reovirus. While the potential link between piscine reovirus and the disease heart and skeletal muscle inflammation is still under scientific debate, this disease had caused mortality of large numbers of salmon in the farm in the month prior to the fishermen's observations. These analyses provide new insights into interactions between domesticated and wild fish.

## Introduction

One of the most significant environmental challenges associated with the commercial culture of Atlantic salmon (*Salmo salar* L.) in marine net pens is containment. Within Norway, where statistics for the number of reported escapees are recorded by the Norwegian Directorate of Fisheries (NDF), the annual numbers of escapees has been in the hundreds of thousands for most years in the period 2000–2011 [1]. However, the true annual number of escapees has been estimated to be in the millions due to underreporting [2]. Farmed escapees can disperse over long distances [3], [4], may enter rivers [5], and can display a range of ecological [6] and genetic interactions [7]–[12] with wild conspecifics. Thus, it is generally accepted that farmed escapees represent a potential threat to the integrity of native populations.

The application of molecular-genetic methods for wildlife conservation and fisheries management purposes, including forensic cases for law enforcement and regulation is expanding [13]. Typical wildlife forensic applications range from species identifications for morphologically unidentifiable tissues and samples, to population of origin identifications for individuals suspected to have been taken from locations where harvest is regulated or illegal [14], or even falsely claimed [15]. Analysis of stomach and faeces content from predators has also been extensively conducted, and provided identification of prey items at the species [16]–[20], family [21], and even individual sample level [22].

The NDF are responsible for the development and implementation of aquaculture regulation in Norway. While escapement of fish from commercial aquaculture installations is not illegal in Norway, farmers are legally bound to report escapement from their farms. Despite this, underreporting represents a major challenge faced by the NDF. In response to this situation, genetic methods for the identification of escapees back to their farm of origin have been established and resulted in fines for companies found in breach of regulations [23], [24].

**Figure 1:**
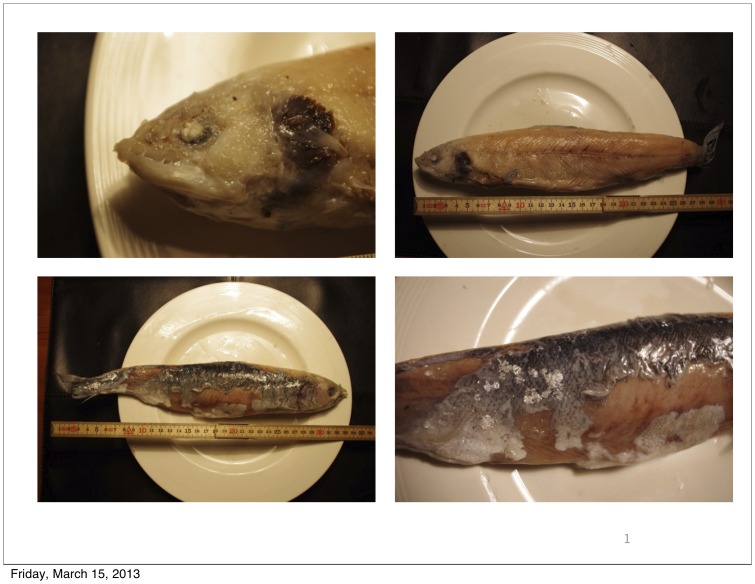
Examples of prey sampled from Atlantic cod stomachs. Most of the 36 prey were more severely digested than the specimens presented here and morphologically impossible to identify. However, not all prey were photographed.

In March 2012, local fishermen operating in a fjord in Northern Norway reported catching Atlantic cod (*Gadus morhua* L.), which forms an important commercial fishery in this region, with unusual prey fish in their stomachs. Most of these prey that were approximately 30–35 cm long, were partially or heavily degraded, and as such it was challenging to identify all of them morphologically (Fig. 1). Nevertheless, they did not look like herring (*Clupea harengus* L.) or smaller gadoid species which form an important part of the cod's diet in this region [25], [26], and it was speculated by several fishermen that these could be Atlantic salmon. While Atlantic cod have been known to ingest Atlantic salmon smolts upon migration from freshwater into estuarine and marine environment [27], [28], within a few weeks of entering the marine environment in the late spring and early summer, smolts have typically left fjord areas and migrate towards oceanic feeding grounds. As such, cod ingesting wild salmon of the observed size and time of year at this location was considered unusual by the local fishermen, and the situation therefore reported to the NDF as a suspected interaction between a local salmon farm and this commercial fishery. Here, we report the analysis of the prey in order to address the following questions: 1. What species are these prey, 2. If they are salmon, is it possible to identify them as wild or farmed (i.e., is this a rare natural phenomena or is it a human induced), and 3. If they are farmed Atlantic salmon, did they originate from a local farm?

## Materials and Methods

### Methodological approach

The present study was designed to address the three questions presented in the introduction. Diagnostic markers for identification of severely degraded Atlantic salmon and rainbow trout (*Oncorhynchus mykiss*) tissues have been recently developed [29]. However, the first attempt at identification of the prey was conducted with highly polymorphic microsatellite markers commonly implemented in Atlantic salmon population genetics projects. The reasoning for this was two-fold. First, in order to answer questions 2 and 3, an allele-frequency profile would be needed for each of the prey in order to match against the allele frequency profiles of farmed and wild salmon in the region. Second, a combination of past experiences with these microsatellite markers on partially degraded samples, together with inspection of the prey suggested that if they were indeed Atlantic salmon, it may be possible to successfully genotype the samples with these microsatellites.

### Samples

This study is based in a fjord located in Northern Norway. For legal reasons, the exact locations of the cod captured in this study, and the local fish farm from which samples were taken, remain anonymous. Under supervision of the NDF, a total of 36 prey were sampled from cod stomachs by local fishermen (1–3 prey per cod stomach, all cod captured in the period March to April 2012). These cod were captured as part of a commercial harvest and were dead upon their stomachs being sampled. Thus, no specific permits were required for sampling the cod stomachs in this study. Both North east Arctic cod (NEAC) and Norwegian coastal cod (NCC) are known to form the basis of this commercial fishery at this time of the year in this region. However, no samples of nor data were recorded from these cod and as such it is not possible to exclude these fish of either type.

All cod were captured at one of six locations in the immediate vicinity of the only fish farm in the region containing salmon overlapping in size with the prey, or up to a maximum of 20 km further in the fjord. In addition to the 36 prey captured in cod stomachs, a single salmon post smolt, captured in the monitoring net located immediately beside the only salmon farm in this region, was sampled (this individual fish is hereafter referred to as “the escapee” and was similar in size to the prey). From all of these samples, two tissue samples per individual were taken for later genetic analysis.

Samples of salmon from the only farm in this fjord that contained fish overlapping in size with the prey were also collected. These fish were sampled by persons employed at the NDF. No specific permits were required to sample these fish, although the fish farmer gave access to their farm. The nearest alternative farm rearing fish overlapping in size with the prey was located over 100 km away (120 km away for the most distant captures of the cod) and not seen as a likely source, and therefore not sampled. From the local farm, a total of three samples, each consisting of approximately 47 fish, were taken from three separate cages. This represented the three genetic groups of fish on the farm, and is consistent with the sampling protocol for establishing a genetic baseline for identification of escapees back to their farm of origin [23], [24].

A sample of wild Atlantic salmon, originating from the nearest river and in the immediate vicinity to where the cod with prey in their stomachs were captured was also included in this study. This wild salmon sample consisted of 101 adults captured by angling in the river in the seasons of 2007 and 2008. As these fish were captured and subsequently killed for consumption by sports fishermen, no permits for taking scale samples from these dead fish were required.

### Disease status on the farm

Heart and skeletal muscle inflammation (HSMI) is an infectious disease [30] characterized by extensive inflammation and multifocal necrosis of myocytes in heart and red musculature [31]. A novel virus, piscine reovirus (PRV) has recently been detected in fish with HSMI. This virus is associated with the disease, shows elevated viral load in diseased fish, and is potentially responsible for the disease [32], [33]. However, PRV infections are common in farmed salmon in Norway, and has also been documented in healthy fish including wild salmon [34]. Therefore, the role of PRV in HMSI remains under debate [34].

In the period January to February 2012 (i.e., a few weeks prior to the discovery of salmon-like prey in the stomachs of wild cod), the local farm reported losses of approximately 55000 fish (data from NDF farm biomass register). The causative disease was subsequently diagnosed as HMSI in February 2012. This diagnosis was based upon clinical analyses of fish from the farm by a local veterinary officer, and was subsequently confirmed by the Norwegian Veterinary Institute using histopathology (therefore, the presence or absence of PRV in these diseased fish is unknown).

Due to the background information regarding the disease status on the farm, samples from the prey captured in cod stomachs, and the single escapee, were analysed for the presence of PRV. This was on the basis that PRV could be present in the prey and the escapee if they originated from the farm where HMSI had caused mortality. PRV is also present in wild Norwegian salmon (also in fish not displaying HMSI), albeit at a lower frequency than in farmed escaped salmon (13.4% vs. 55.2% prevalence respectively) [34]. While this virus is typically identified in heart or head-kidney samples, due to the degraded state of the prey, only muscle samples were available for this test. Analyses were conducted by a Real Time PCR analysis company, PatoGen Analyse AS, accredited according to international standard ISO 17025. The samples were analyzed for PRV RNA at PatoGen in accordance with their in-house methods for Real Time PCR using an assay (‘PRV-ST’) targeting the L3 gene, sequenced previously [32]. The sequences of the forward and reverse primers for this assay are 5′-TCAACCACCTCCACACAAAAGA-3′ and 5′-AACGAGTTGTGCGTGTGCC-3′ respectively, and the probe VIC-5′-TTGGGATGTCGACGTTCT-3′. The standard curve based on tenfold dilutions in triplicates had a slope of −3.25 (R^2^ = 0.998), and the Efficiency (E =  [10^1/(–slope)^] – 1) was 1.030. The cut-off C_T_ value was 37.0. The PRV-ST analysis was not accredited at the time of analysis and this work represents the first time these markers, produced by PatoGen AS, have been published.

### Molecular genetic analyses

DNA extraction was conducted in 96-well format using a commercially available kit (Qiagen DNeasy®96 Blood & Tissue Kit). Each 96-well plate included two blank wells as negative controls. Routine genotyping control plays a standard role in genotyping in the laboratory at IMR [35], [36]. Thus, each of the individual prey and the escapee were isolated twice to control genotyping consistency. DNA quantity and quality was not measured.

All samples were subject to genotyping with a set of 18 microsatellites that are used in the laboratory for Atlantic salmon genetics projects. These loci were amplified in three multiplexes, using standard protocols for fresh tissues (full genotyping conditions available from authors upon request); *SSsp3016* (Genbank no. AY372820), *SSsp2210*, *SSspG7*, *SSsp2201*, *SSsp1605*, *SSsp2216* [37], *Ssa197*, *Ssa171*, *Ssa202* [38], *SsaD157*, *SsaD486*, *SsaD144* [39], *Ssa289*, *Ssa14* [40], *SsaF43* [41], *SsaOsl85* [42], *MHC I* [43] and *MHC II* [44]. PCR products were analysed on an ABI 3730 Genetic Analyser and sized by a 500LIZ™ size-standard. The raw data was controlled manually twice before export for statistical analysis. No genotyping inconsistencies were observed among these re-analysed samples.

### Statistical analysis

Once a DNA profile was successfully established for the individual prey, the single escapee, the local farm and wild salmon from a population in the region, several statistical tests, commonly implemented in population genetics studies, were conducted on these data. This was in order to primarily address three questions posed in the introduction. For these tests, the single escapee was pooled with the prey fish based upon pilot analysis documenting it to be genetically very similar to the prey (see results). Thus, for these analyses, the prey sample also included the single escapee.

First, the data were arranged in a population genetics program (MSA) [45], which was used to compute a range of summary statistics, and input files for other programs. Thereafter, the data was analysed in Genepop V3.3 [46] to compute gene diversities, Hardy Weinberg equilibrium, and linkage disequilibrium between pairs of loci within samples. The Fishers exact test (demorization 10 000; 100 batches; 5000 iterations) was implemented to test for statistical significance. The program LDNE [47] as used to compute the effective population size (*Ne*) for each of the samples. This program uses a one-sample approach to estimating *Ne* based upon the degree of LD observed within a sample.

Genetic identification of the prey was conducted by two different but complimentary methodological approaches. First, genetic assignment using the Rannala & Mountain method of computation [48] as implemented in the program GeneClass2 [49] was conducted. Here, the samples from the farms and of the wild fish were used as the pre-determined potential sources of the prey (i.e., the genetic baseline). Thereafter, direct genetic assignment was conducted. This method places each unknown fish (i.e., the individual prey fish) into the baseline sample that it resembles most. A limitation with direct assignment is that it assigns a potential source population to each of the unknown samples irrespective to the absolute degree of similarity. This may be acceptable in “closed systems” where all potential sources of the unknown samples are represented, however, in situations such as the present where not all potential sources are included in the baseline, it is important to get an estimation of the degree of similarity between the unknown sample(s) and each baseline sample. This is achieved by exclusion, and each individual is compared to each baseline sample, and a probability of belonging (or more correctly, probability of not belonging) is computed. In the specific situation here, rejection from all baseline samples would suggest that the prey originated from a source not sampled.

The second approach to identifying the prey was to compute admixture (also referred to as Bayesian cluster analysis) using the program STRUCTURE 2.2 [50], [51]. Individual admixture permits the identification and assignment of individual fish to genetic clusters (i.e., populations or genetic groups) without any “prior” regarding the population or location from which each individual sample originated. This permits, for example, identification of individuals that are of mixed genetic origin, and identification of individuals when mixed into samples predominantly of other genetic groups. The program was run using an admixture model with correlated allele frequencies and no prior. Runs consisted of a burn-in of 250 000 MCMC steps, followed by 250 000 steps. The program was run with all samples detailed included, with the number of populations set between *k* = 1–8 with 3 runs per *k*. The probability of the data was plotted, and the most appropriate *k* was determined at the point where the slope reached a plateau [50].

## Results

Despite being partially digested (Fig. 1), microsatellite DNA profiles were successfully obtained from all 36 prey sampled from the cod stomachs, and the single escapee captured in the monitoring net placed outside the local farm. While some markers were not scored in some of the DNA isolations, when combining data from both isolates (after cross-checking to validate genotyping consistency), only two genotypes were missing from a total of 629 potential genotypes for the 37 fish analysed at 17 microsatellite loci (i.e., >99% genotyping coverage). This provided both conclusive evidence that the prey were indeed Atlantic salmon, and permitted the next step of their identification using a population genetics statistical approach.

Summary statistics for the combined sample of the prey (which included the single escapee captured in net), samples from the local farm and from the river demonstrate several trends (Table 1). All samples from the farm displayed less genetic diversity than the sample from the river, as measured by either the total number of alleles, or allelic richness which circumvents the problems of having different numbers of individuals representing each sample. Lower variation at polymorphic genetic markers is typical for farm samples in comparison with wild samples [52], [53], and is linked to the fact that the fish sampled in a single cage often have a limited number of parents [54]. Almost all samples were in HWE, however, LD was observed in both the sample Farm 1C, and the sample of prey fish. *Ne* was very low in the sample of the prey and two of the samples from farms. In contrast, *Ne* was much higher in the sample of wild salmon and farm sample 1B. For all of these summary statistics, the prey resembled the farm samples very strongly, especially 1C, whereas they displayed very different parameters to the wild sample.

**Table 1:** Summary statistics for samples from a local farm the group of escapees, and a local wild population.

Sample	N	Gene diversity			HWE		LD		Allelic diversity		Ne
		Ho	He	Fis	0.05	0.001	0.05	0.001	At	Ar	
Farm 1a	47	0.79	0.77	−0.026	0	0	17	1	156	151	43 (36–53)
Farm 1b	46	0.79	0.77	−0.027	1	0	12	0	157	152	125 (84–225)
Farm 1c	46	0.75	0.75	0.002	1	0	18	4	143	139	25 (21–30)
Prey-fish	37	0.76	0.75	−0.016	0	0	30	9	145	145	28 (24–35)
Wild	101	0.79	0.80	0.013	0	0	20	1	236	199	169 (135–222)

N =  number of samples analysed, Ho and He  =  observed and expected heterozygosity, Fis  =  inbreeding coefficient, HWE  =  number of deviations from Hardy Weinberg equilibrium at two significance levels, LD  =  observed linkage disequilibrium at two significance levels, At  =  total number of alleles observed over 17 polymorphic loci, Ar  =  allelic richness based upon a re-sample size of 36–37 per locus/population combination and then totaled over all loci, Ne  =  effective population size as estimated by the LDNA method [47], with 95% confidence intervals in brackets and based upon including alleles down to and including those with frequencies of 0.005 in each population.

F_ST_ is an average measurement of genetic similarity between groups of samples or populations. Taken collectively, the prey were genetically strongly distinct to the wild salmon, and marginally different to the samples farm 1A and 1B (Table 2). These prey were genetically similar to the farm sample 1C. All farm samples were genetically distinct to the wild sample, supporting observations from the summary statistics presented above.

**Table 2:** Genetic relationships among the sets of samples as measured by pair-wise F_ST_ (data in upper right diagonal), with associated P-values (data in lower left diagonal).

Sample	Farm1A	Farm 1B	Farm 1C	Prey	Wild
Farm1A		0.002	0.010	0.013	0.057
Farm 1B	0.144		0.006	0.006	0.052
Farm 1C	0.0008	0.0131		0.001	0.064
Prey	0.0008	0.0102	0.26		0.070
Wild	0.0001	0.0001	0.0001	0.0001	

Self-assignment simulations including samples from the farm and the wild population demonstrated that overall, 70% of the fish in this set of samples would be correctly assigned to the sample from which they originated. Miss-assignment was caused almost exclusively by farmed fish being incorrectly placed into an alternative farm sample which reflects the overlapping genetic profile between these cages. None of the farmed fish were incorrectly assigned to the wild population, and only 3 of the 101 wild salmon were incorrectly assigned to any of the farmed samples (all were assigned to Farm 1B). Thus, these simulations demonstrate almost complete potential to identify whether the prey are more likely to have originated from this local farm (and thus a human-induced event) or the local wild population (and therefore an unusual natural event).

**Figure 2:**
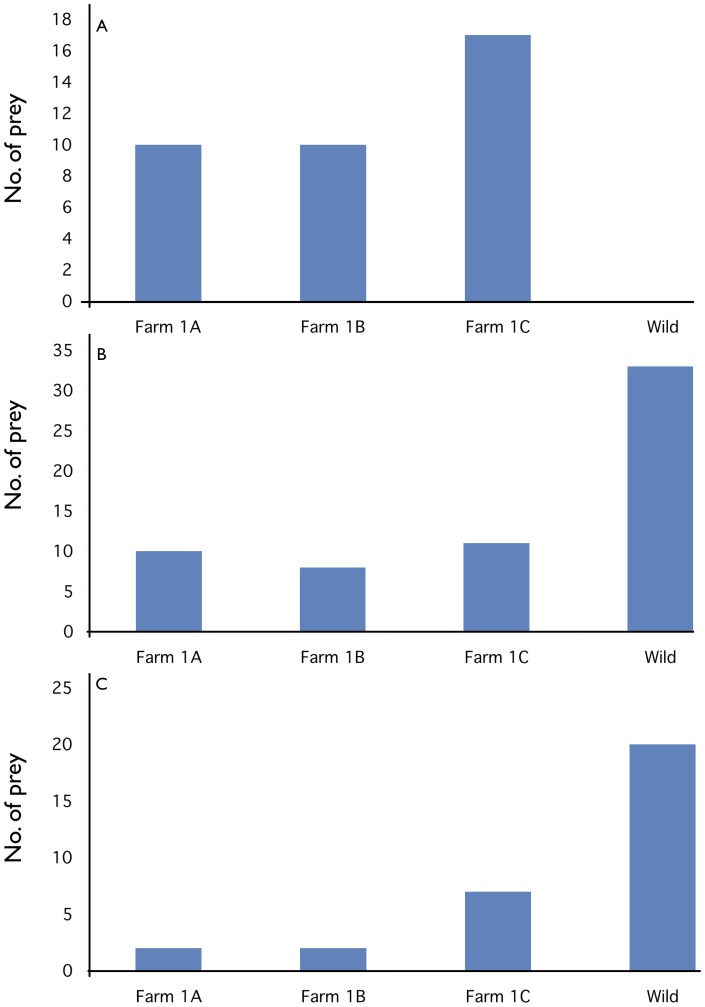
Genetic assignment of the prey to the samples collected from a local Norwegian farm and to the nearest wild Atlantic salmon population. A =  direct assignment of prey to the genetically most similar sample, B =  exclusion of prey from each sample in turn at α 0.01 threshold, C =  exclusion of prey from each sample in turn at α 0.001 threshold. Note that individual prey can in theory be excluded from all or none of the samples, thus, exclusion does not sum to the exact number of prey in contrast to direct assignment which adds up to 37.

Direct assignment (which places the unknown sample, which is in this case was the 36 prey and the one salmon escapee captured in a net outside the farm, into the genetically most similar baseline sample) placed all of the prey and the single escapee into farm samples, and none into the wild sample (Fig. 2). Exclusion tests supported this, demonstrating that the majority of the prey and the single escapee could be conclusively excluded from the sample of wild salmon, whilst only 1 prey sample could be excluded from all of the farmed samples (and in that case the wild sample also).

**Figure 3:**
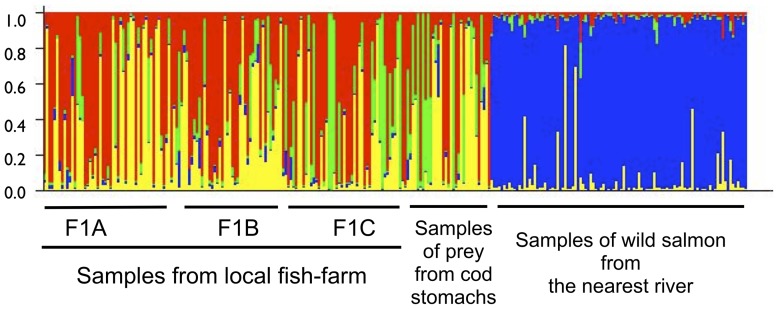
Admixture analysis of salmon representing fish collected from the local Norwegian farm, prey captured in cod stomachs, and the nearest wild Atlantic salmon population. Results of admixture analysis are presented when the number of genetic clusters (i.e., *k*) is set to 4. Each genetic cluster is represented by a colour, and each individual's genetic assignment is represented by a vertical bar. Individuals may be admixed (i.e., mixtures of genetic clusters).

Identification of the prey and the single escapee was also conducted using admixture analysis in the program Structure (Fig. 3). This program does not take into consideration any “priors” for the samples and each individual can represent a mixture of genetic groups or clusters. The probability of the data was plotted, and the most appropriate number of clusters *k*, was determined to be 4 (the point where the slope reached a plateau) [50]. Confirming results from other statistical tests presented above, admixture analysis demonstrated that there was a large genetic difference between the farmed salmon and the wild salmon in this data set, and importantly, that all of the prey, including the single salmon escapee, were closely associated with genetic clusters represented in the salmon from the local Norwegian farm, and not the local wild population. Data for other numbers of clusters (i.e., *k* set between 2–8) gave identical results (data not presented).

Real-time PCR analyses (PRV-ST assay) detected PRV virus RNA in 22 of the 37 prey samples. The positive samples represented 21 prey items from cod stomachs and the single salmon captured in the monitoring net placed outside the Norwegian farm. C_T_ values ranged from 27.8–35.3 (mean 33.0).

## Discussion

Molecular-genetic tools to identify the aquaculture facility and in some cases even the specific cage of origin for Atlantic salmon [23], [24], Atlantic cod [55], [56] and farmed rainbow trout [57] escapees have been developed. However, the present study represents a new application of molecular-genetic methods in order to provide management authorities with the opportunity to monitor commercial aquaculture and its interaction with the natural environment. In addition, this study provides new insights into interactions between domesticated and wild fish.

Four main conclusions can be drawn from these analyses: 1. The partially digested and morphologically difficult to identify prey were revealed to be Atlantic salmon, 2. Based upon several independent genetic parameters, these salmon prey were identified as farmed and not from the local wild population, thus demonstrating this to be a human induced, as opposed to natural phenomena, 3. Despite partial digestion, the majority of the prey, including the single escapee, carried detectable levels of PRV. PRV is associated with the disease HSMI [32], [33]. This disease had caused significant mortality of salmon on the local farm in the immediate time-period prior to the prey being captured in the wild cod, 4. The genetic profile of the salmon prey, and the single escapee, strongly matched the genetic profile of the fish in the local farm. Although genetic similarity is not unequivocal proof of origin [23], considering the nearest alternative farm that these individuals could have theoretically originated from was located over 100 km away in another fjord, these analyses provided the NDF with sufficient “circumstantial evidence” to initiate an investigation of the company owning this commercial aquaculture facility on the basis of potential mis-management.

The salmon farm in the study area was diagnosed with HSMI just weeks prior to the appearance of farmed salmon in the stomach of the local cod. Therefore, the prey recaptured from cod stomachs were examined for the presence of PRV, although virus levels may decline after an outbreak [58]. Despite partial digestion of the prey, and the fact that only muscle samples were available, PRV virus was still detected. Nevertheless, based upon the analyses conducted here, it is not possible to unequivocally resolve how the PRV infected farmed salmon entered the natural environment. They could be diseased dead fish deposited into the sea (which would represent an illegal practice in Norway) and thereafter ingested by the cod from the sea-bed, or they were escapees predated upon by the cod. Given that the farm had experienced significant mortality of fish (55000) through HMSI in the period immediately before the salmon were captured in the cod stomachs, indicate that the former explanation is the most likely.

Independent of how the fish entered the natural environment, this study demonstrates trophic transmission as a mechanism for interaction between salmon farming and wild populations. While Atlantic cod have been documented to predate upon wild Atlantic salmon smolts migrating from freshwater to the sea [27], [28], Atlantic salmon is not typically predated upon by cod at the time of year in which the current study was conducted [25], [26]. Furthermore, to our knowledge, this study represents the first documentation of Atlantic cod ingesting Atlantic salmon from a fish farm. Thus, it is possible that the cod investigated here, and forming part of the population in the study area at this time of year, have been exposed to PRV. The ability of PRV virus to be transmitted to new hosts via ingestion of infected prey is at present unknown, as is the susceptibility of Atlantic cod to the virus. However, PRV was not detected in 78 cod nor 850 other gadoids that were recently sampled in Norway and screened for this virus [59]. Since PRV infections are common in wild and farmed salmon in Norway, and also occur in wild sea trout (*Salmo trutta* L.) [34], it is likely that the common PRV type is specific to salmonids and that cod is not susceptible.

Analysis of animal stomach contents or faeces using molecular genetic methods has been widely applied to a range of taxa and biological questions [19], [60]. These methods have primarily been conducted to identify prey items to a taxonomic classification, often species, usually involving the analysis of a single or low number of genes providing the required taxonomic capability for the potential prey species in question [17], [18], [20]. More recently, advances in next generation sequencing have permitted powerful additions to these approaches, leading to what is termed as DNA metabarcoding [61]. While the present study does not represent a technological advance for such molecular genetic methods, the application of microsatellite DNA analysis in diet analysis to provide identification beyond a taxonomic classification is novel. Here, it was possible to not only demonstrate that the prey was Atlantic salmon, but that the most probable source was a local farm and not a local wild salmon population. Finally, it was also possible to demonstrate that the prey carried a virus that has been associated with a disease that causes significant mortality of farmed salmon. Therefore, this study represents an extension of the biological questions that can be addressed via molecular genetic analysis of stomach contents. Other examples of diet analysis going beyond species identifications include analysis to the family level to demonstrate filial cannibalism in the wild [21], and predation mortality of Atlantic salmon of farmed, hybrid and wild parentage in a natural river system (Skaala unpublished). Also, identification of prey items to the individual level has been conducted, where microsatellite analysis of diet from greenland sharks (*Somniosus microcephalus*), together with a search of a DNA register for all minke whales (*Balaenoptera acutorostrata*) captured under commercial harvest in Norway [62] permitted connecting the whale and shark captures in both time and space to understand both their movement and diet habits [22].

For more than a decade, the annual reported escapement of salmon from Norwegian fish farms has been in the tens or hundreds of thousands [1]. This is likely to be an underestimate due to underreporting, and in the period 1998 to 2004, it is estimated that the mean annual number of escapees was 2.4 million [2], which is higher than the annual number of wild salmon returning to the Norwegian coastline to reproduce in the same period. While attention surrounding the impact of escapees has primarily been given to those alive as opposed to dead [7], [9], [63], the present study demonstrates that virus-infected farmed fish may be released to the environment by one method or another. The analyses in the present case highlight the potential to identify and track such events. Given the magnitude of escapement from commercial fish farms, this represents one of the most significant human-induced invasions of native populations by a species that has been subject to selective breeding. Therefore, this situation needs to be monitored for not only ecological [6] and genetic [7]–[12] interactions, but also disease interactions.
